# Effect of Rosmarinic Acid on the Serum Parameters of Glucose and Lipid Metabolism and Oxidative Stress in Estrogen-Deficient Rats

**DOI:** 10.3390/nu11020267

**Published:** 2019-01-25

**Authors:** Maria Zych, Ilona Kaczmarczyk-Sedlak, Weronika Wojnar, Joanna Folwarczna

**Affiliations:** 1 Department of Pharmacognosy and Phytochemistry, School of Pharmacy with the Division of Laboratory Medicine in Sosnowiec, Medical University of Silesia, 40-055 Katowice, Poland; isedlak@sum.edu.pl (I.K.-S.); wwojnar@sum.edu.pl (W.W.); 2 Department of Pharmacology, School of Pharmacy with the Division of Laboratory Medicine in Sosnowiec, Medical University of Silesia, 40-055 Katowice, Poland; jfolwarczna@sum.edu.pl

**Keywords:** rosmarinic acid, estrogen-deficient rats, metabolic disorders, oxidative stress

## Abstract

Rosmarinic acid is found in medicinal and spice plants such as rosemary, lemon balm, and mint. The aim of the study was to investigate the effect of rosmarinic acid on parameters of glucose and lipid metabolism and parameters of oxidative stress in rats in the early phase of estrogen deficiency. The study was carried out on mature female Wistar rats divided into the following groups: sham-operated control rats, ovariectomized control rats, and ovariectomized rats treated orally with rosmarinic acid at a dose of 10 mg/kg or 50 mg/kg daily for 28 days. The concentration of sex hormones, parameters related to glucose and lipid metabolism as well as parameters of antioxidant abilities and oxidative damage were determined in the blood serum. In the ovariectomized control rats, the homeostasis model assessment of insulin resistance (HOMA-IR) index and cholesterol concentration increased, the superoxide dismutase activity increased, and the reduced glutathione concentration decreased. Administration of rosmarinic acid at both doses induced decreases in the fructosamine concentration and HOMA-IR, an increase in the concentration of reduced glutathione, and a decrease in the concentration of advanced oxidation protein products in ovariectomized rats. Moreover, rosmarinic acid at a dose of 50 mg/kg induced a decrease in the total cholesterol and triglyceride concentrations. The results indicate that rosmarinic acid may be useful in the prevention of metabolic disorders associated with estrogen deficiency, however further studies are necessary.

## 1. Introduction

Estrogen deficiency leads to numerous metabolic disorders, including, among others, adverse changes in lipid profile and insulin resistance [1,2]. The consequence of these disorders is the development of cardiovascular diseases, which are the cause of 49% of deaths in women in Europe [3]. Observations from the 1990s suggested that the use of hormone replacement therapy (HRT) in postmenopausal women prevents cardiovascular diseases [4,5], but later studies conducted as part of the Women’s Health Initiative study indicated the connection between HRT and an increased risk of venous thromboembolism, stroke, coronary heart disease and breast cancer [6]. There is an interest in the search of compounds which could replace HRT as its safer alternative.

Recently, the growing popularity of so-called functional food has been observed. Despite providing nutritional benefits, this kind of food may affect physiological processes in the body, for example cardiovascular system health. Nutraceuticals, which are products containing concentrated active substances derived from food, are also popular. Postmenopausal women use food and supplements containing phytoestrogens of the isoflavone group [7,8,9]. Phenolic acids are a group of compounds with potential preventive and therapeutic properties in lipid and carbohydrate metabolism disorders [10,11]. These compounds also exhibit antioxidant activity [12]. Until now it has been shown that phenolic acids, such as sinapic acid, ferulic acid or caffeic acid, which are derivatives of hydroxycinnamic acid, beneficially affect disorders of lipid profile and/or glucose homeostasis in different experimental rodent models [13,14,15,16,17,18], including estrogen-deficient rats [17,18].

Rosmarinic acid, a derivative of hydroxycinnamic acid, occurs mainly in plants of the Lamiaceae family, which are widely used as spices and medicinal plants in traditional Western (*Rosmarinus officinalis* L., *Melissa officinalis* L., *Mentha*×*piperita* L., *Salvia officinalis* L., *Thymus vulgaris* L.) and Chinese (*Perilla frutescens* (L.) Britton, *Salvia miltiorrhiza* Bunge, and *Rabdosia rubescens* (Hemsl.) H. Hara) medicine [19,20]. Rosmarinic acid has been reported to exert, among others, antioxidant, anti-inflammatory, neuroprotective, and cardioprotective activities [19,20,21]. There are reports of favorable effects of rosmarinic acid on glucose and lipid metabolism in different experimental models of diabetes in male rats [22,23,24,25,26]. It should be stressed that there are sex-specific differences in glucose and lipid metabolism [27,28]. Taking into account the National Institute of Health statement on the lack of balance in the use of animals of both sexes in experimental studies (predominance of studies on males) [29], it seems important to evaluate the effect of rosmarinic acid on glucose and lipid metabolism in female rats.

The aim of the study was to investigate the effect of rosmarinic acid at doses of 10 and 50 mg/kg daily for four weeks on parameters of glucose and lipid metabolism and oxidative stress in the early phase of estrogen deficiency in rats. The bilaterally ovariectomized rats used in the study, in which the administration of rosmarinic acid started one week after the surgery, constituted a model of early postmenopause pre-diabetic changes.

## 2. Materials and Methods

### 2.1. Experimental Design

The study was carried out with the approval of the Local Ethics Committee in Katowice (permission numbers: 38/2015, 148/2015 and 66/2016). Three-month old Wistar female rats, purchased from the Center of Experimental Medicine, Medical University of Silesia (Katowice, Poland) were used in the experiment. During acclimatization period (13 days) and throughout the experiment, the rats had unrestricted access to drinking water and standard feed (Labofeed B, Wytwórnia Pasz Morawski, Kcynia, Poland), and were kept in standard conditions, complying with the European Union guidelines (directive 2010/63/EU). The animals were divided into 4 groups (n = 10): sham-operated control rats (SHAM); ovariectomized control rats (OVX); ovariectomized rats treated with rosmarinic acid at a dose of 10 mg/kg daily (OVX + RA10); ovariectomized rats treated with rosmarinic acid at a dose of 50 mg/kg daily (OVX + RA50). Rats from the OVX, OVX + RA10 and OVX + RA50 groups underwent bilateral ovariectomy, while the sham-operated rats underwent sham surgery. Both ovariectomy and sham surgery were performed under general anesthesia induced by intraperitoneal (i.p.) injection of a mixture of ketamine (Ketamina 10%, Biowet Puławy, Puławy, Poland) and xylazine (Xylapan, Vetoquinol Biowet, Gorzów Wlkp., Poland). This experiment shared controls (both SHAM and OVX) with our previously reported study [18]. The administration of rosmarinic acid (Sigma-Aldrich, St. Louis, MO, USA) to the animals from groups OVX+RA10 and OVX+RA50, and water to the animals from groups SHAM and OVX started seven days after the surgery. Rosmarinic acid was dissolved (10 mg/kg) or suspended (50 mg/kg) in tap water with the addition of Tween 20 (up to 1 µL/1 mL tap water; Sigma-Aldrich, St. Louis, MO, USA) and administered in a volume of 2 mL/kg. Rosmarinic acid or tap water with the same amount of Tween 20 (control rats) were administered to rats orally (p.o.–per os) once daily for 28 days by an intragastric tube. All animals were weighed twice a week. Before the last administration of rosmarinic acid or vehicle, the final measurements of the body mass were performed.

On the next day after the last administration of rosmarinic acid or water, after overnight fasting, the rats were anesthetized with ketamine and xylazine and sacrificed by cardiac exsanguination. The uterus, thymus, liver and right kidney were isolated and weighed. The blood was used to obtain the serum, which was frozen until the biochemical parameters were determined. All spectrophotometric measurements were performed using a Tecan Infinite M200 PRO plate reader with Magellan 7.2 software (Tecan Austria, Grödig, Austria). 

### 2.2. Determination of Serum Concentrations of Estradiol and Progesterone

ELISA kits produced by DiaMetra (Segrate-Milano, Italy) were used to determine the serum estradiol and progesterone concentrations, according to the manufacturer’s instructions.

### 2.3. Determination of Serum Concentrations of Glucose, Insulin and Fructosamine, and HOMA-IR Index

The glucose concentration was determined by a Pointe Scientific (Canton, MI, USA) kit, while the insulin concentration was determined by means of a BioVendor ELISA (Brno, Czech Republic) kit. To calculate the HOMA-IR index (homeostasis model assessment of insulin resistance), the following formula was used:

HOMA-IR = (fasting glucose (mg/dL) × fasting insulin (μU/mL))/405(1)

The concentration of fructosamine was determined spectrophotometrically using a Pointe Scientific (Canton, MI, USA) kit.

### 2.4. Determination of Serum Concentrations of the Total Cholesterol, Low-Density Lipoprotein Cholesterol, High-Density Lipoprotein Cholesterol and Triglycerides

To determine the concentration of total cholesterol, low-density lipoprotein cholesterol (LDL-C), high-density lipoprotein cholesterol (HDL-C) and triglycerides, Pointe Scientific (Canton, MI, USA) kits were used. The determinations were made according to the manufacturer’s instructions.

### 2.5. Determination of Serum Concentrations of Reduced and Oxidized Glutathione, and Total Antioxidant Capacity

The total concentration of glutathione (TotGSH) and the concentration of oxidized glutathione (GSSG) were determined using a Cayman Chemical (Ann Arbor, MI, USA) kit. The concentration of reduced glutathione (GSH) was calculated according to the formula: GSH = TotGSH − 2 × GSSG (nmol/mL), and then the GSH/GSSG ratio was determined. Total antioxidant capacity (TAC) was also determined using a Cayman Chemical (Ann Arbor, MI, USA) kit and following the manufacturer’s instructions.

### 2.6. Determination of Serum Activities of Superoxide Dismutase and Catalase

Cayman Chemical (Ann Arbor, MI, USA) kits were used to determine the activities of antioxidant enzymes: superoxide dismutase (SOD) and catalase (CAT). The activity of SOD and CAT was expressed in U or nmol/min, respectively, per 1 mg of serum protein. The serum protein content was determined by means of the biuret method, using a Pointe Scientific (Canton, MI, USA) kit. All measurements were carried out following the instructions provided by manufacturers. 

### 2.7. Determination of Serum Concentrations of Thiobarbituric Acid Reactive Substances, Advanced Products of Protein Oxidation and Protein Carbonyl Groups

The concentration of thiobarbituric acid reactive substances (TBARS) was determined spectrophotometrically using the method of Ohkawa et al. [30]. This method is based on the reaction between lipid peroxidation products and thiobarbituric acid. The intensity of the color obtained during this reaction was measured at 535 nm. The standard curve was prepared from 1,1,3,3-tetraethoxypropane (Sigma-Aldrich, St. Louis, MO, USA).

The concentrations of advanced oxidation protein products (AOPP) was determined spectrophotometrically according to Witko-Sarsat et al. [31]. Chloramine T (Sigma-Aldrich, St. Louis, MO, USA) was used to establish the calibration curve, the absorbance was measured at 340 nm. The concentration of AOPP was expressed in nmol/mL of the chloramine T equivalents. 

The concentration of protein carbonyl groups (PCG) was measured spectrophotometrically using a Cell Biolabs (San Diego, CA, USA) kit, according to the instructions of the manufacturer.

### 2.8. Determination of Serum Concentrations of Interleukin 18, Uric Acid, Urea and Creatinine, and Activities of Aspartate Aminotransferase and Alanine Aminotransferase

An ELISA kit from Cloud-Clone (Houston, TX, USA) was used to determine the concentration of interleukin 18 (IL-18). The concentrations of uric acid and urea, as well as the activities of aspartate aminotransferase (AST) and alanine aminotransferase (ALT) were determined spectrophotometrically, using kits from BioSystems (Costa Brava, Barcelona, Spain). A Pointe Scientific (Canton, MI, USA) kit was used to determine the creatinine concentration. The instructions of the manufacturers were followed.

### 2.9. Statistical Analysis

The results are presented as the arithmetic mean ± standard error of the mean (SEM). One-way ANOVA and Fisher’s LSD post-hoc test were used to assess statistical significance of the results (Statistica 12 software, StatSoft Polska, Kraków, Poland). It was assumed that the results are statistically significant if *p* < 0.05. Moreover, those results, which differed from the results of the control rats at *p* < 0.06 (Student’s *t*-test) were described as trends in the text.

## 3. Results

### 3.1. Effect of Rosmarinic Acid on the Body Mass, Body Mass Gain, Mass of Selected Organs and Serum Concentrations of Sex Hormones

**Figure 1:**
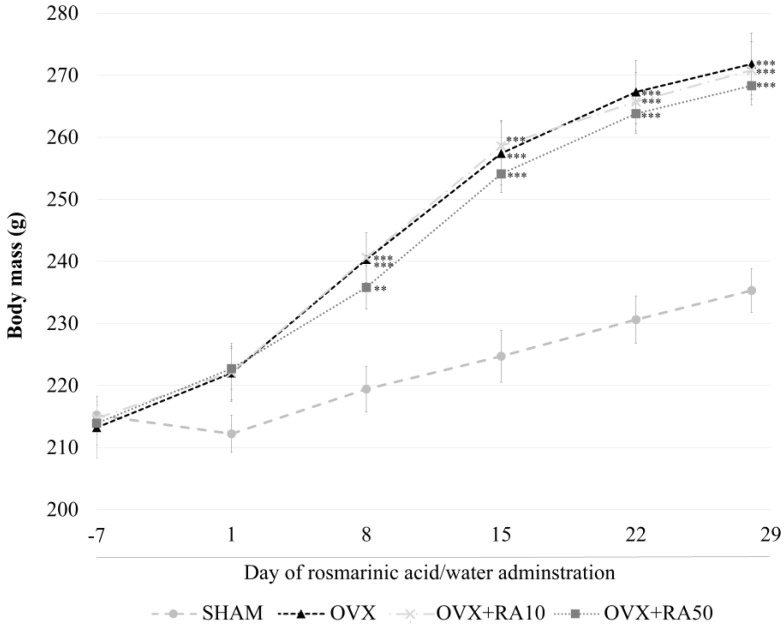
Effect of rosmarinic acid on the body mass in ovariectomized rats. Rosmarinic acid at doses of 10 mg/kg (OVX + RA10) and 50 mg/kg (OVX + RA50) was administered orally to ovariectomized rats, once daily for 28 days. SHAM: sham-operated control rats; OVX: ovariectomized control rats. The final measurements of the body mass were made before the last rosmarinic acid or water administration (28th day). Results are presented as the mean ± SEM. One-way ANOVA followed by Fisher’s LSD test were used for evaluation of the significance of the results. ** *p* < 0.01, *** *p* < 0.001: significantly different from the SHAM control rats.

**Figure 2:**
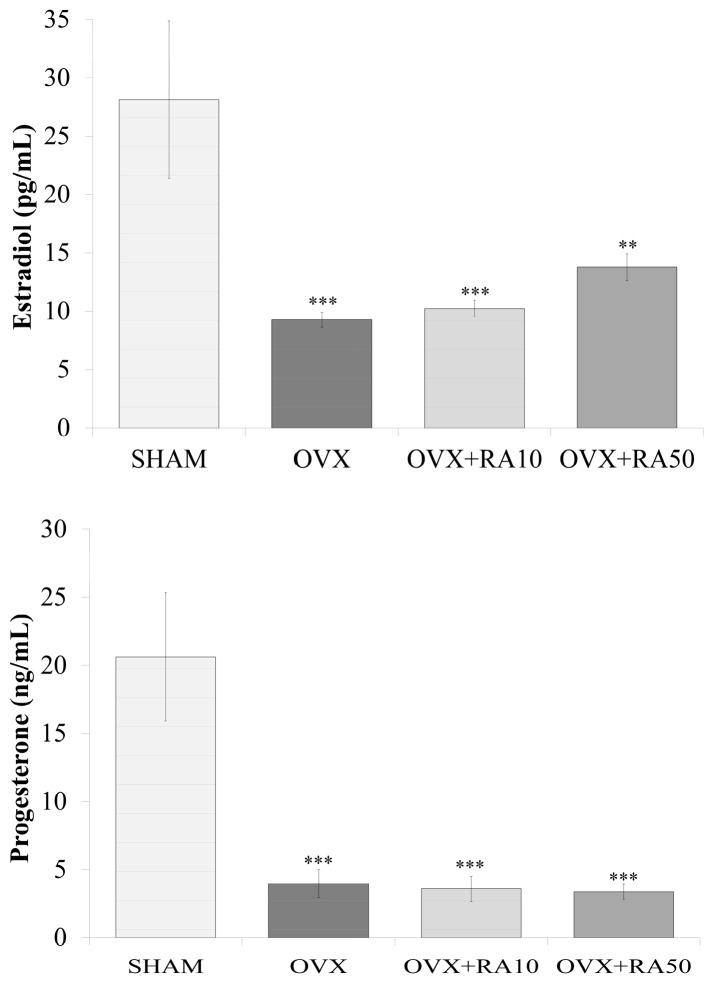
Effect of rosmarinic acid on the serum concentration of estradiol and progesterone in ovariectomized rats. Rosmarinic acid at doses of 10 mg/kg (OVX + RA10) and 50 mg/kg (OVX + RA50) was administered orally to ovariectomized rats, once daily for 28 days. SHAM: sham-operated control rats; OVX: ovariectomized control rats. Results are presented as the mean ± SEM. One-way ANOVA followed by Fisher’s LSD test were used for evaluation of the significance of the results. ** *p* < 0.01, *** *p* < 0.001: significantly different from the SHAM control rats.

As was previously reported [18], estrogen deficiency induced by ovariectomy caused a statistically significant increase in the body mass (Figure 1) and body mass gain, a decrease in the uterine mass and an increase in the thymus mass, without affecting the liver and kidney mass compared to the sham-operated control rats (data not shown). There was also a decrease in the serum estradiol and progesterone levels (Figure 2). Administration of rosmarinic acid at doses of 10 mg/kg and 50 mg/kg to estrogen deficient rats did not affect the body mass (Figure 1), body mass gain and the mass of the internal organs compared to the ovariectomized control rats (data not shown). Rosmarinic acid administered at a dose of 10 mg/kg to ovariectomized rats did not affect the estradiol and progesterone concentrations in comparison with the ovariectomized control rats, whereas after administration of rosmarinic acid at a dose of 50 mg/kg, the concentration of estradiol showed an increasing trend. There was no effect on progesterone concentration (Figure 2). 

### 3.2. Effect of Rosmarinic Acid on the Serum Concentrations of Glucose, Insulin and Fructosamine, and the HOMA-IR Index

**Figure 3:**
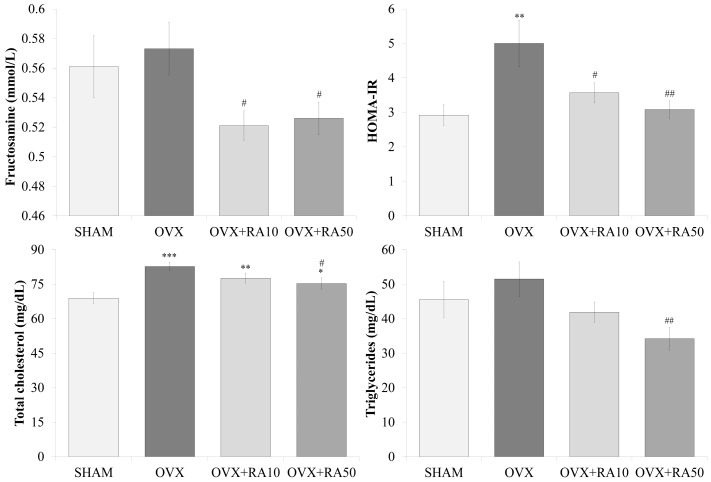
Effects of rosmarinic acid on the selected serum parameters related to glucose and lipid metabolism in ovariectomized rats. Rosmarinic acid at doses of 10 mg/kg (OVX + RA10) and 50 mg/kg (OVX + RA50) was administered orally to ovariectomized rats, once daily for 28 days. SHAM: sham-operated control rats; OVX: ovariectomized control rats; HOMA-IR: homeostasis model assessment of insulin resistance. Results are presented as the mean ± SEM. One-way ANOVA followed by Fisher’s LSD test were used for evaluation of the significance of the results. * *p* < 0.05, ** *p* < 0.01, *** *p* < 0.001: significantly different from the SHAM control rats. # *p* < 0.05, ## *p* < 0.01: significantly different from the OVX control rats.

As was reported [18], no significant effects on the serum concentrations of glucose, insulin (data not shown) and fructosamine (Figure 3) were observed in rats 5 weeks after the ovariectomy, although the values of those parameters were slightly increased in relation to the sham-operated controls. However, insulin resistance assessed by the HOMA-IR index significantly increased (Figure 3). After using both doses of rosmarinic acid, the fructosamine concentration and the HOMA-IR index significantly decreased compared to the ovariectomized control rats. Rosmarinic acid administered to estrogen-deficient rats at doses of 10 mg/kg and 50 mg/kg did not significantly affect the glucose and insulin levels (data not shown). 

### 3.3. Effect of Rosmarinic Acid on the Serum Concentrations of Total Cholesterol, LDL-C, HDL-C and Triglycerides

As a result of the administration of rosmarinic acid at a dose of 50 mg/kg, the total cholesterol concentration slightly but significantly decreased compared to the ovariectomized control rats, in which estrogen deficiency induced significant increases in the total cholesterol and LDL-C concentrations in relation to the sham-operated control rats (Figure 3, the results for LDL-C and HDL-C not shown). The triglyceride level significantly decreased in comparison with the ovariectomized controls after administration of rosmarinic acid at the higher dose. Rosmarinic acid at 10 mg/kg did not significantly affect those lipid metabolism parameters in estrogen-deficient rats. 

### 3.4. Effect of Rosmarinic Acid on the Serum Concentrations of GSH, GSSG and TAC

**Figure 4:**
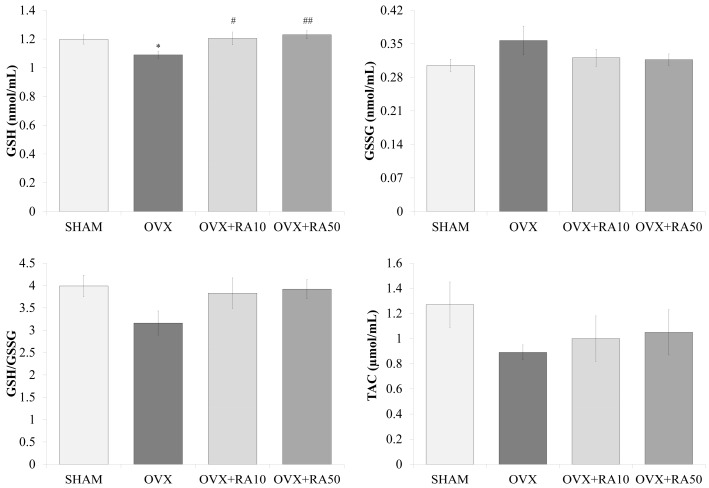
Effect of rosmarinic acid on the serum concentrations of reduced glutathione (GSH), oxidized glutathione (GSSG), GSH/GSSG ratio and total antioxidant capacity (TAC). Rosmarinic acid at doses of 10 mg/kg (OVX + RA10) and 50 mg/kg (OVX + RA50) was administered orally to rats once daily for 28 days. SHAM: sham-operated control rats; OVX: ovariectomized control rats. Results are presented as the mean ± SEM. The level of TAC is presented in Trolox equivalents. One-way ANOVA followed by Fisher’s LSD test were used for evaluation of the significance of the results. * *p* < 0.05: significantly different from the SHAM control rats. # *p* < 0.05, ## *p* < 0.01 – significantly different from the OVX control rats.

Rosmarinic acid, administered to ovariectomized rats at both doses of 10 and 50 mg/kg induced significant increases in GSH concentration, which was reduced due to estrogen deficiency. The concentration of GSSG did not significantly change either as a consequence of estrogen deficiency or due to the administration of rosmarinic acid. There was a trend to decrease the GSH/GSSG ratio in the ovariectomized control rats in comparison to the sham-operated control rats, and a trend to increase the GSH/GSSG ratio in rats administered rosmarinic acid at a dose of 50 mg/kg in relation to the ovariectomized control rats. There was no significant effect of rosmarinic acid on TAC in ovariectomized rats (Figure 4).

### 3.5. The Effect of Rosmarinic Acid on the Serum Activities of SOD and CAT, and Concentrations of TBARS, AOPP and PCG

Rosmarinic acid at both doses (10 and 50 mg/kg) did not affect the SOD and CAT activities; the former was increased due to estrogen deficiency. The use of rosmarinic acid at both 10 mg/kg and 50 mg/kg resulted in a statistically significant reduction in the AOPP concentration, but did not significantly affect the PCG concentration, compared to the ovariectomized control rats. There was a trend to decrease the TBARS concentration after administration of rosmarinic acid at a dose of 10 mg/kg to the ovariectomized rats, while the higher dose did not significantly affect this parameter when compared to the ovariectomized control rats. It should be noted that estrogen-deficiency did not induce significant changes in AOPP, PCG and TBARS levels in relation to the sham-operated control rats (Table 1).

### 3.6. Effect of Rosmarinic Acid on the Serum Concentrations of Interleukin 18, Uric Acid, Urea and Creatinine, and the Activities of AST and ALT

In estrogen deficient rats, a trend to decrease the IL-18 concentration was observed in relation to the sham-operated control rats. The use of rosmarinic acid did not significantly affect the IL-18 serum level. Rosmarinic acid administered to ovariectomized rats did not change the uric acid, urea or creatinine concentrations (data not shown). Rosmarinic acid at both used doses did not significantly affect the AST and ALT activities in ovariectomized rats (Table 2).

## 4. Discussion

Oxidative stress is believed to be associated with the reduction in estrogen levels in the postmenopausal period [32]. The most commonly used method to monitor oxidative stress is the measurement of parameters of antioxidant abilities and oxidative damage in the serum, since it is the least invasive and easily available method. It was found that the serum concentrations of oxidative damage markers (e.g., TBARS, AOPP, PCG) were higher, and the serum indices of antioxidant abilities parameters (e.g., GSH) were lower in postmenopausal women than in premenopausal women [33,34,35]. In addition, the glucose and lipid metabolism disorders are observed in postmenopausal women [35,36]. 

The animal model which is commonly used to study the effects of different agents on metabolic disorders induced by estrogen deficiency is a model of bilateral ovariectomy in rats [37,38,39]. In the present study, the measurements were performed 5 weeks after the ovariectomy. Since 5 weeks in adult rats correspond to about 3.3 years in humans [40], the model was supposed to mirror the changes in early years after menopause in women. In this model, changes in the bone and lipid metabolism were demonstrated [17,41,42]. It should be stated that development of significant effects on carbohydrate metabolism require longer periods of estrogen deficiency [43]. Taken together, the used model may be described as a pre-diabetic early postmenopause rat model.

Consistent with previous reports [39,44], the body mass gain was significantly higher, and the serum estradiol and progesterone concentrations were significantly lower in ovariectomized rats in comparison to the non-ovariectomized control rats. Estrogen deficiency contributed to the changes in circulating antioxidant parameters and oxidative damage indicators. As was previously reported [18], in the control ovariectomized rats, the SOD activity increased, while the GSH concentration decreased. No statistically significant changes in the concentrations of TBARS, PCG and AOPP were noted. Since significant increases in malondialdehyde (MDA), which is the main component of TBARS, were demonstrated 12 weeks, but not 4 and 8 weeks after ovariectomy [45], it is possible that the 5-week period of estrogen deficiency was not long enough to increase significantly the oxidative stress parameters in the ovariectomized control rats. Nevertheless, in the ovariectomized control animals, the total cholesterol and LDL cholesterol levels, as well as the HOMA-IR index, increased compared to the control rats with normal estrogen levels [18]. The increase in the HOMA-IR, and the lack of significant effects on the insulin and glucose concentrations, indicate that the rats 5 weeks after the ovariectomy were pre-diabetic.

Due to the fact that oxidative stress is associated with metabolic disorders, it is supposed that substances with antioxidant activity may be useful in their prevention and treatment [46]. One such antioxidative agent is rosmarinic acid. 

In this study, rosmarinic acid was administered to ovariectomized rats at doses of 10 and 50 mg/kg p.o. daily for 4 weeks. Rosmarinic acid is found in lemon balm, rosemary or mint in the amount of 7.1–27.4 mg/g of dry matter [47,48]. Herbs containing rosmarinic acid are often used in self-healing and daily diet, so it is possible to consume 5-10 g of these herbs per day in the form of infusions and as spices [49,50,51]. Rosmarinic acid is soluble in water, and, according to literature data, extraction efficiency of this compound in infusions is around 90% [48]. Therefore, it is possible to consume about 110 mg of rosmarinic acid daily, i.e. about 1.6 mg/kg for adult humans weighing 70 kg. The lower dose used in rats in this study (10 mg/kg) corresponds to the amount of rosmarinic acid that can be consumed by humans in the form of spices, herbal teas and infusions, taking into account the conversion factor of 6.17 resulting from faster metabolism in rats [52]. The higher dose was chosen in order to investigate whether the dose 5 times higher than the dose achievable in the diet may exert a stronger therapeutic effect.

In the present study, rosmarinic acid favorably influenced parameters related to the redox balance measured in the serum. Even though the use of rosmarinic acid did not affect enzymatic antioxidant indicators (SOD and CAT), it induced an increase in the GSH concentration. GSH is the main non-enzymatic antioxidant that regulates redox homeostasis. An increase in the plasma GSH concentration due to the use of rosmarinic acid was previously described in various models of diabetes [22,53]. It was shown that rosmarinic acid stimulates up-regulation of catalytic subunits of glutamate cysteine ligase (an enzyme involved in the biosynthesis of GSH) in hepatic stellate cells [54]. On the other hand, another in vitro study [55] demonstrated an unfavorable (inhibitory) effect of rosmarinic acid on enzymes involved in the regeneration of GSH from GSSG (glutathione reductase and glucose-6-phosphate dehydrogenase). Thus, it can be assumed that the increase in the GSH concentration after rosmarinic acid administration, observed in the present study, was rather a result of intensified biosynthesis of GSH than of its restoration from the oxidized form. Moreover, it should be noted that rosmarinic acid after absorption in rats occurs mostly as its metabolites [56]. It is possible that these metabolites may also play a role in the observed increase in the GSH concentration. Furthermore, the serum GSH/GSSG was calculated, since it is known to be an important indicator for the cellular redox state, and also for the redox state on the tissue and whole body levels [57]. The beneficial effect of rosmarinic acid on redox homeostasis was shown by a trend to increase the GSH/GSSG ratio in the rat serum. 

Rosmarinic acid also improved the oxidative damage parameters in the serum of ovariectomized rats. After its administration at both doses, there was a decrease in the AOPP concentration and after the lower dose—a trend to decrease the TBARS level in relation to the ovariectomized controls, consistent with a report on the effects of rosmarinic acid in diabetic rats [22]. Since lipid peroxidation products such as MDA may lead to protein and DNA damage [58], and AOPP can promote reactive oxygen species (ROS) formation via receptor for advanced glycation end products (RAGE)-dependent pathway [59], the influence of rosmarinic acid on the oxidative damage parameters observed in the present study suggests its beneficial, health-promoting effect. The rosmarinic acid effect may be especially valuable in later stages of estrogen deficiency when oxidative stress may be more increased. It can be speculated that the reduction of the formation of AOPP by rosmarinic acid results from the inhibition of myeloperoxidase activity. Myeloperoxidase is an enzyme that catalyzes the production of hypochlorous acid (a factor inducing the formation of AOPP), in the reaction of chloride ion with hydrogen peroxide [60]. Rosmarinic acid was demonstrated to decrease the activity of myeloperoxidase in the cerebral cortex, kidneys and pancreas [61,62,63].

Moreover, rosmarinic acid and its metabolites may directly neutralize ROS [19] and thereby reduce the formation of oxidative damage products. The antioxidant activity of rosmarinic acid results directly from its structure, more precisely from the presence of 4 hydrogens in the phenolic system, and two catechol moieties, that give this compound a polar character. Electrochemical studies have shown that rosmarinic acid is oxidized in two steps. In the first step, the caffeic acid moiety is oxidized, and in the second step, the 3,4-dihydroxyphenyl lactic acid residue. Therefore, rosmarinic acid is considered to be the strongest antioxidant of all hydroxycinnamic acid derivatives [19].

In the present study, we observed that administration of rosmarinic acid led to a decrease in the fructosamine concentration in the pre-diabetic ovariectomized rats. Although there was no significant effect of estrogen deficiency on the fructosamine level in the control rats, the decreasing effect of rosmarinic acid may indicate that this acid prevents the non-enzymatic glycation of proteins, as fructosamine is the product of the reaction of glucose carbonyl group with the amino groups of serum proteins. The inhibition of the formation of advanced glycation end-products under the influence of rosmarinic acid has been previously presented in vitro and in vivo [26,64]. Administration of rosmarinic acid at both doses reduced the HOMA-IR index in comparison to the ovariectomized control rats. The improvement of the insulin sensitivity by rosmarinic acid was previously demonstrated in rats in various diabetes models [22,24,25]. As demonstrated in previous experimental studies, the beneficial effects of rosmarinic acid on glucose metabolism may result from an increased glucose uptake in skeletal muscle cells via activation of adenosine monophosphate-activated kinase (AMPK) and increased glucose transporter 4 (GLUT4) translocation [24,25,65]; increased insulin secretion from pancreatic islets [66], and increase in the expression of GLUT2 in the pancreas [66] or modulating the trafficking of sodium-glucose cotransporter 1 (SGLT1) to the enterocyte brush-border membrane [67]. Moreover, rosmarinic acid exerted beneficial effects on the expression of the hepatic genes or proteins involved in insulin signaling, and glucose and lipid metabolism, such as insulin receptor substrate-1 (IRS-1), AMPK, phosphoenolpyruvate carboxykinase (PEPCK), GLUT2, forkhead box protein O1 (FOXO1), sterol regulatory element-binding protein 1 (SREBP1) and carnitine palmitoyltransferase 1 (CPT1) in diabetic rats [23,25]. A possible mechanism of rosmarinic acid effect on glucose and lipid metabolism may be the peroxisome proliferator-activated receptor γ (PPARγ)-mediated action; rosmarinic acid was reported to activate those receptors [68]. Thiazolidinediones, which also are PPARγ agonists, exert antidiabetic action, primarily based on the normalization of lipid metabolism in adipose tissue [69]. 

The use of rosmarinic acid in the present study resulted in a decrease in the serum levels of triglycerides and total cholesterol as compared to the ovariectomized control rats; statistically significant changes were observed after administration of the higher dose (50 mg/kg). The beneficial effect of rosmarinic acid on the lipid profile has already been described in experimental models of diabetes [23,26] and acute myocardial infarction [70] in rats. Although, based on our research, we cannot explain the mechanism of the serum lipid reducing action of rosmarinic acid in our experimental model, it is known from the literature data that rosmarinic acid inhibited the expression of SREBP1 (which activates genes involved in fatty acid and triglyceride synthesis) in insulin resistant HepG2 cells. In addition, in insulin resistant HepG2 cells, rosmarinic acid induced the expression of CPT1, which controls the entry of fatty acids into the mitochondria for β-oxidation [23]. Rosmarinic acid is the ester of caffeic acid and 3-(3,4-dihydroxyphenyl)lactic acid, and, in the rat body, is partially metabolized to coumaric acid and caffeic acid [56]; the hypolipidemic effect of rosmarinic acid could also result from the action of its metabolites. For example, caffeic acid inhibited hepatic fatty acid synthase, 3-hydroxy-3-methylglutaryl CoA reductase and acyl-CoA:cholesterol acyltransferase activities, and increased fatty acid β-oxidation activity in high-fat diet-induced-obese mice [14]. In our previous studies, we demonstrated a reduction in the serum concentration of total cholesterol after administration of caffeic, p-coumaric and chlorogenic acids to ovariectomized rats [17], and decreases in the serum concentrations of total cholesterol and triglycerides as a result of sinapic acid administration [18]. Both caffeic acid and sinapic acid increased the serum estradiol concentration in estrogen-deficient rats, which might have contributed to the observed metabolic effects [17,18].

In the present study, the rosmarinic acid did not cause a significant increase in the serum estradiol concentration in estrogen deficient rats, but a higher dose (50 mg/kg) induced a trend to increase the estradiol levels. Since in our previous study caffeic acid increased the serum estradiol concentration in ovariectomized rats [17], the slight increase in the estradiol concentration observed in the present study may be a result of the action of rosmarinic acid metabolites. In ovariectomized rats, the extra-ovarian tissues such as adipose tissue, skin, bones and brain, are the source of estradiol. In those sites, C19 steroids cannot be synthetized; it is only possible to convert C19 steroids (androgens) to estrogens by aromatase [71]. Therefore, it seems possible that rosmarinic acid or its metabolites increase aromatase activity. Although there are reports that aromatase was inhibited by some polyphenols [72,73], the increases in the serum estradiol concentration after using polyphenol-rich products, such as dried pomegranate concentrate powder and black tea extract, have already been described in ovariectomized rats [74,75]. It should be pointed out that caffeic acid increased the estradiol and decreased total cholesterol concentrations only in rats fed a standard diet containing soy [17], and those effects were not observed in rats fed a soy-free chow with lowered content of phenolic acids [41]. Therefore, it is possible that at least some of the demonstrated effects of rosmarinic acid may depend on the diet.

In the present study, rosmarinic acid exerted a similar beneficial influence on some lipid parameters and insulin resistance (HOMA-IR) as that demonstrated for sinapic acid in a parallel study [18]. While the metabolic effects of sinapic acid appeared to be rather estrogen-dependent [18], it seems that, in case of rosmarinic acid, its antioxidant activity may play a more important role. It should be noted that the lower dose of rosmarinic acid (10 mg/kg) was sufficient to decrease the HOMA-IR index and fructosamine concentration, while the higher dose (50 mg/kg) was necessary to decrease the total cholesterol and triglyceride levels in estrogen-deficient rats. 

In our study, the observed favorable effects of rosmarinic acid on the parameters associated with glucose and lipid metabolism in female rats in the early phase of estrogen deficiency were slight, whereas earlier studies showed a stronger effect of rosmarinic acid in various models of diabetes in male rats [22,23,25,26]. Although differences in the potency of rosmarinic acid may suggest a dependence of its effect on sex, we cannot confirm this thesis based on our results. The results of our experiment (on rats with slight pre-diabetic changes) and the results of studies carried out in models of established diabetes cannot be directly compared. Moreover, there were differences in doses of rosmarinic acid used, as well as in the duration of its administration (100 mg/kg p.o. for 30 days [22,23]; 120, 160 and 200 mg/kg i.p. for 7 or 28 days [25]; 30 mg/kg p.o. for 8 weeks [26]).

One limitation of the study is that it evaluated the effect of rosmarinic acid on the investigated parameters at one time-point (5 weeks after ovariectomy) only. The other limitation is that we focused only on measurements of oxidative stress parameters to elucidate the mechanism of effects of rosmarinic acid on the serum biochemical markers of carbohydrate and lipid metabolism. Further studies are necessary, including, among others, hepatic expression of proteins involved in insulin signaling, lipogenesis and lipolysis in estrogen-deficient rats. 

## 5. Conclusions

Rosmarinic acid favorably affected the HOMA-IR index and certain parameters of lipid metabolism in pre-diabetic early postmenopause model in rats, exerting some antioxidant activity. It seems that rosmarinic acid may be useful in the prevention of metabolic disorders associated with estrogen deficiency, however further studies are necessary.

## Figures and Tables

**Table 1:** Effect of rosmarinic acid on the serum activity of antioxidative enzymes and concentrations of oxidative damage markers in ovariectomized rats.

Parameter/Group	SHAM	OVX	OVX + RA10	OVX + RA50
SOD (U/mg of protein)	5.22 ± 0.19	6.20 ± 0.34 *	6.39 ± 0.35 **	6.21 ± 0.27 *
CAT (nmol/min/mg of protein)	0.52 ± 0.08	0.74 ± 0.19	0.74 ± 0.14	0.91 ± 0.25
TBARS (nmol/mL)	21.5 ± 1.9	24.1 ± 1.9	19.1 ± 0.7	19.9 ± 1.1
AOPP (nmol/mL)	19.9 ± 1.2	25.0 ± 4.4	16.9 ± 2.0 ^#^	13.6 ± 2.2 ^##^
PCG (nmol/mL)	5.52 ± 0.40	5.87 ± 0.85	5.16 ± 0.44	5.00 ± 0.95

Rosmarinic acid at doses of 10 mg/kg (OVX + RA10) and 50 mg/kg (OVX + RA50) was administered orally to ovariectomized rats, once daily for 28 days. SHAM: sham-operated control rats; OVX: ovariectomized control rats; SOD: superoxide dismutase; CAT: catalase; TBARS: thiobarbituric acid reactive substances; AOPP: advanced oxidation protein products; PCG: protein carbonyl groups. Results are presented as the mean ± SEM. The concentration of AOPP is presented in chloramine T equivalents. One-way ANOVA followed by Fisher’s LSD test was used for evaluation of the significance of the results. * *p* <0.05, ** *p* <0.01: significantly different from the SHAM control rats. # *p* <0.05, ## *p* <0.01—significantly different from the OVX control rats.

**Table 2:** Effect of rosmarinic acid on the serum concentration of interleukin 18, and activity of aspartate aminotransferase (AST) and alanine aminotransferase (ALT) in ovariectomized rats.

Parameter/Group	SHAM	OVX	OVX + RA10	OVX + RA50
Interleukin 18 (pg/mL)	312.0 ± 13.7	235.0 ± 22.7	298.0 ± 27.3	315.9 ± 33.5
AST (U/L)	39.22 ± 3.38	34.72 ± 2.78	37.60 ± 3.57	41.61 ± 2.57
ALT (U/L)	23.68 ± 2.19	23.52 ± 1.38	25.20 ± 1.86	30.65 ± 3.84

Rosmarinic acid at doses of 10 mg/kg (OVX+RA10) and 50 mg/kg (OVX+RA50) was administered orally to rats, once daily for 28 days. SHAM: sham-operated control rats; OVX: ovariectomized control rats. Results are presented as the mean ± SEM. One-way ANOVA followed by Fisher’s LSD test were used for evaluation of the significance of the results.
